# Regulation of the *Pseudomonas syringae* Type III Secretion System by Host Environment Signals

**DOI:** 10.3390/microorganisms9061227

**Published:** 2021-06-05

**Authors:** Megan R. O’Malley, Jeffrey C. Anderson

**Affiliations:** Department of Botany and Plant Pathology, Oregon State University, Corvallis, OR 97331, USA; omallmeg@oregonstate.edu

**Keywords:** *Pseudomonas syringae*, type III secretion system (T3SS), plant pathogens, plant–microbe interactions, host–pathogen signaling, bacterial pathogenesis, two-component systems

## Abstract

*Pseudomonas syringae* are Gram-negative, plant pathogenic bacteria that use a type III secretion system (T3SS) to disarm host immune responses and promote bacterial growth within plant tissues. Despite the critical role for type III secretion in promoting virulence, T3SS-encoding genes are not constitutively expressed by *P. syringae* and must instead be induced during infection. While it has been known for many years that culturing *P. syringae* in synthetic minimal media can induce the T3SS, relatively little is known about host signals that regulate the deployment of the T3SS during infection. The recent identification of specific plant-derived amino acids and organic acids that induce T3SS-inducing genes in *P. syringae* has provided new insights into host sensing mechanisms. This review summarizes current knowledge of the regulatory machinery governing T3SS deployment in *P. syringae*, including master regulators HrpRS and HrpL encoded within the T3SS pathogenicity island, and the environmental factors that modulate the abundance and/or activity of these key regulators. We highlight putative receptors and regulatory networks involved in linking the perception of host signals to the regulation of the core HrpRS–HrpL pathway. Positive and negative regulation of T3SS deployment is also discussed within the context of *P. syringae* infection, where contributions from distinct host signals and regulatory networks likely enable the fine-tuning of T3SS deployment within host tissues. Last, we propose future research directions necessary to construct a comprehensive model that (a) links the perception of host metabolite signals to T3SS deployment and (b) places these host–pathogen signaling events in the overall context of *P. syringae* infection.

## 1. *Pseudomonas syringae* Is a Type III Secretion System-Producing Plant Pathogen

*Pseudomonas syringae* are Gram-negative γ-proteobacteria [1,2] that infect and cause disease in plants, in many cases significantly impacting plant health and agricultural yield [3,4]. The *P. syringae* species complex includes more than 50 isolated pathogenic strains referred to as pathovars [5,6]. Individual pathovars of *P. syringae* are typically capable of infecting only a narrow range of plant species, though collectively *P. syringae* cause disease on a diverse range of terrestrial plants [7]. Due to their impact on the health of various commercially relevant agricultural and ornamental plant species, *P. syringae* are ranked among the most damaging and costly of agricultural plant pathogens [8].

*P. syringae* are ubiquitous in the environment and generally survive in soil, plant debris, and water at various stages of its cycle, i.e., in groundwater, freshwater bodies, clouds, and precipitation [7]. Some *P. syringae* pathovars are also able to persist epiphytically on the aerial surfaces of plants for extended periods of time [9]. *P. syringae* are often spread from infected plant tissue or environmental reservoirs to nearby healthy plant hosts by wind and/or rain splash, and can also be transmitted vertically through infected seeds [9,10]. Once established on a host plant, *P. syringae* invade the intercellular spaces (or apoplast) of aboveground plant tissue, often finding ingress through openings such as stomata or surface wounds. *P. syringae* can rapidly multiply to high population levels within the host apoplast, leading to the formation of disease symptoms such as necrotic, water-soaked lesions or yellowing (chlorosis) of infected tissue. These symptoms may negatively impact the vigor and productivity of infected plants, in some cases leading to significant crop loss [9,11]. Diseases caused by *P. syringae* are often controlled by preventative measures such as the sterilization of seedstock and conventional breeding of disease-resistant plant cultivars, as well as disease management strategies that include the application of bactericidal or bacteriostatic compounds [3,4].

### 1.1. The P. syringae Type III Secretion System (T3SS) and Its Role in Virulence

*P. syringae* rely on a type III secretion system (T3SS) to cause disease in host plants [12,13]. The T3SS is a syringe-like translocon comprised of a barrel-like basal body that spans both bacterial cell membranes, and an extracellular filament termed the pilus [14,15]. The T3SS pilus extends through the plant cell wall and likely makes direct contact with the host plasma membrane, forming a continuous channel between the bacterium and host cell. While the T3SS is a common feature among both plant and animal pathogens, plant pathogens including *P. syringae* produce a T3SS pilus that is significantly longer than those typically produced by animal pathogens, presumably in order to breach the thick barrier of the plant cell wall [14,16].

During host infection, a subset of bacterial proteins, collectively termed T3SS effectors, are trafficked through the hollow inner channel of the T3SS and delivered directly into the host cytosol. Following their translocation into the host cell, many T3SS effectors inhibit various facets of host immune signaling, effectively suppressing the host defense response [17]. *P. syringae* pathovars typically possess a repertoire of >20 different effectors, which carry out various functions within the plant host [18,19,20,21]. Some T3SS effectors directly interact with and disrupt the function of plant cell-surface receptors and/or co-receptors that are involved in the immune detection of microbial invaders [22,23,24]. Furthermore, numerous *P. syringae* T3SS effectors interrupt various stages of the host signaling cascade initiated by these immune receptors, and/or curtail the deployment of immune-triggered antimicrobial defenses [25,26,27,28]. Other T3SS effectors promote pathogen entry into the apoplast by regulating stomatal aperture, through interaction with plant immune regulators such as RIN4 or the modulation of the plant hormone jasmonic acid [29,30,31]. Additionally, T3SS effectors can promote *P. syringae* fitness in the apoplast by stimulating water release from host cells [32].

**Figure 1:**
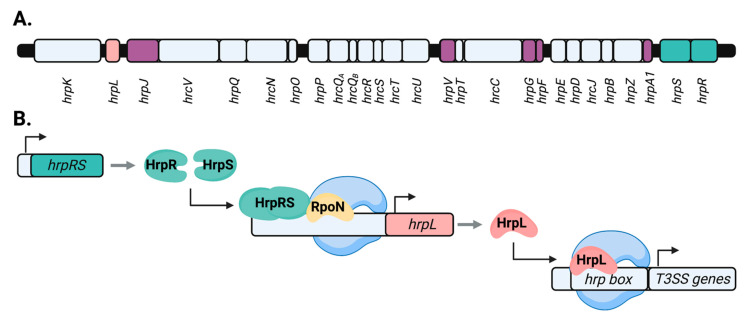
Regulation of the T3SS by components of the *hrp/hrc* pathogenicity cluster in *P. syringae*. (**A**) Depicted is a schematic of the *hrp/hrc* pathogenicity cluster within the tripartite T3SS pathogenicity island in *P. syringae* pv. *syringae* 61. This schematic is adapted from Alfano et al., 2000 [34]. Colored boxes depict open reading frames, with corresponding *hrp/hrc* gene names listed below. Genes encoding T3SS master regulators HrpL and HrpRS are shaded in coral and teal, respectively. Shaded in violet are additional regulators of T3SS expression that function at either the transcriptional or post-transcriptional level. (**B**) Depicted is a diagram of the T3SS regulatory cascade in *P. syringae*. Genes encoding the regulatory proteins HrpR and HrpS (teal) are transcribed from the *hrpRS* operon within the *hrp/hrc* pathogenicity cluster. HrpRS oligomerize (depicted as a dimer for clarity) and bind the *hrpL* promoter. Together with alternate sigma factor RpoN (gold), which recruits RNA polymerase (blue) to the *hrpL* promoter, HrpRS stimulate transcription of *hrpL*. HrpL (coral) functions as an alternate sigma factor to recruit RNA polymerase to a conserved binding motif, termed the “hrp box,” in the promoters of target genes. By this mechanism, HrpL directs the transcription of downstream genes, including T3SS-associated genes. Figure was made using BioRender (https://app.biorender.com, accessed on 30 April2021).

Most T3SS effectors contribute redundantly to disarming host immunity and are individually dispensable for virulence. However, individual genes encoding the T3SS translocon itself are required for *P. syringae* to effectively colonize host tissues [12,33,34]. Similarly, while the T3SS effector repertoire varies substantially across different *P. syringae* pathovars, core components of the T3SS are highly conserved throughout Gram-negative bacteria [34,35]. Genetic loci encoding the T3SS were thus designated as *hrp* (*hypersensitive response and pathogenicity*) and/or *hrc* (*hypersensitive response and conserved*) genes, which are organized into the conserved *hrp*/*hrc* pathogenicity cluster (Figure 1A). While many genes within the *hrp/hrc* cluster are required for *P. syringae* virulence, the specific function of several of these virulence factors remains ambiguous [36,37]. For example, the regulatory gene *hrpT* encodes a putative lipoprotein required for T3SS production and virulence by *P. syringae*, yet its function has not been fully determined [36,37,38]. The *hrp/hrc* cluster is located within the tripartite pathogenicity island (T-PAI), along with two loci encoding the majority of T3SS effectors [34]. The canonical T-PAI is present in most lineages within the *P. syringae* species complex, and is thought to be the result of horizontal gene acquisition by an ancestral strain similar to the non-pathogenic soil bacteria *Pseudomonas fluorescens* [17,39]. Additionally, many T3SS effector genes are located outside of the T-PAI throughout the *P. syringae* genome [40].

### 1.2. Transcriptional Control of P. syringae T3SS Genes by hrp/hrc Regulators

The master regulator of the T3SS is HrpL, an extracytoplasmic function (ECF) alternative sigma factor that interacts with a conserved “*hrp*-box” motif in the promoters of target genes (Figure 1B) [41,42,43,44]. By recruiting RNA polymerase, HrpL directs the transcription of the *hrp*/*hrc* pathogenicity cluster as well as various T3SS effector genes [20,21,44,45,46,47,48]. HrpL also regulates the expression of genes unrelated to T3SS function, including those involved in various metabolic processes and the production of bacterial toxins relevant to *P. syringae* pathogenesis [21,44,48,49]. The resolution of the full HrpL regulon in *P. syringae* was enabled by the availability of whole-genome sequences for various *P. syringae* isolates, beginning with model strain *P. syringae* pv. tomato DC3000 [50,51,52]. As the timing and magnitude of induction differ among HrpL-regulated genes, some authors have postulated that HrpL orchestrates the expression of the T3SS-encoding genes in a hierarchical manner [48,53,54]. By this model, genes encoding the core regulatory and structural components of the T3SS apparatus, such as the extracellular pilus protein HrpA1, are initially activated by HrpL, while genes encoding T3SS effectors and secretion accessory proteins are transcribed secondarily [48]. A variety of genes unrelated to T3SS function have also been identified.

The transcription of *hrpL* itself is directed by the σ^54^ sigma factor (RpoN) and the enhancer-binding proteins HrpR and HrpS [43,55,56,57]. The genes encoding HrpR and HrpS are paired in a *hrpRS* operon within the *hrp/hrc* cluster [56,58]. In *P. syringae* pv. *phaseolicola* NPS3121, HrpR is required for *hrpS* transcription, potentially through interaction with an upstream regulatory “HrpR-box” sequence [58]. HrpR and HrpS oligomerize to form a heterohexameric complex and together are required for full *hrpL* gene expression, though HrpS is capable of functioning independently as a weak activator of the *hrpL* promoter in the absence of HrpR [56,59]. In addition to activation of *hrpL*, HrpS directs the expression of a variety of T3SS-independent genes throughout the *P. syringae* genome [45,60,61]. A broad de-repression of housekeeping gene expression has been observed in *hrpL^-^* and *hrpRS^-^* loss-of-function mutants of *P. syringae* pv. tomato DC3000, indicating that a tradeoff between the activation of the T3SS cascade and central cellular metabolism may occur in *P. syringae* [60]. Due to the central role of HrpL and HrpR/HrpS in orchestrating T3SS deployment, the majority of characterized mechanisms of T3SS regulation described in more detail below converge on the activity of these key upstream regulators.

HrpA1, the pilus protein that forms the extracellular filament of the T3SS, also functions as an auto-regulator of T3SS gene expression [14,62]. Early studies of T3SS expression in *P. syringae* pv. tomato DC3000, a pathogen of tomato and the model plant Arabidopsis, noted that HrpA1 was required for the extracellular secretion of T3SS substrates, including harpins and T3SS effectors, as well as the intracellular accumulation of T3SS-encoding mRNA transcripts including upstream regulators *hrpL* and *hrpRS* [62]. HrpA1 was also required for the accumulation of *hrpL* and *hrpS* transcripts by *P. syringae* pv. *phaseolicola* 1448a in the leaves of host bean plants [57]. Interestingly, HrpA1 did not influence *hrpR* transcript abundance in this study, despite the fact that *hrpR and hrpS* are typically co-transcribed in *P. syringae* [56,63]. This effect may be due to the hypothesized role of HrpR as a transcriptional activator of *hrpS* expression in some *P. syringae* pathovars, such as *P. syringae* pv. *phaseolicola* NPS3121 [58]. The mechanism by which HrpA1 positively auto-regulates the expression of the T3SS remains unknown.

In addition to autoactivation by HrpA1, the T3SS cascade is also subject to negative self-regulation. HrpL negatively regulates its own promoter activity through binding to a *hrp*-box element within the promoter of *hrpJ*, a regulatory gene in the HrpL regulon whose promoter is adjacent to that of *hrpL* [54]. Subtle changes in the level of *hrpL* transcripts in *P. syringae* conditioned large-scale shifts in the expression level of downstream T3SS-encoding genes, suggesting that substantial signal amplification occurs through the HrpL–T3SS signaling cascade [54]. As such, negative autogenous regulation of the T3SS cascade may be a way to fine-tune HrpL levels in order to prevent excessive accumulation of T3SS substrates.

### 1.3. Post-Translational Control of the P. syringae T3SS by hrp/hrc Regulators

Additional proteins encoded within the conserved *hrp*/*hrc* pathogenicity cluster also contribute to the regulation of T3SS deployment. HrpS, half of the HrpRS heterodimer that stimulates the transcription of *hrpL*, is subject to post-translational regulation by regulatory pair HrpV and HrpG. By the current model, HrpV directly binds to HrpS, thus limiting the ability of HrpS to bind to HrpR [36,59,64]. HrpV also binds to HrpG, a chaperone protein associated with the T3SS basal body on the cytoplasmic face of the bacterial inner membrane [59,65]. Under conditions where T3SS expression is activated, HrpV is hypothesized to dock at the cell membrane through its interaction with HrpG, thus spatially preventing HrpV association with bacterial DNA and the repression of HrpS [66]. At the inner membrane, the HrpVG complex additionally binds to HrpJ, another regulatory protein encoded in the *hrp/hrc* cluster. HrpJ is secreted through the T3SS, and carries out an unknown function within plant cells to suppress the host immune response [67]. Prior to its own translocation, HrpJ functions to plug the T3SS inner channel through the formation of a gatekeeper-like ternary complex with HrpVG [66]. The formation of this gatekeeper complex may occur via HrpG interaction with HrcU, an additional inner membrane-associated component of the T3SS core [66]. HrpJ is necessary for full T3SS function by *P. syringae* in the host plant environment, and is required for the extracellular secretion of harpins and translocator proteins [67,68,69]. The occlusion of the T3SS inner channel by the HrpVGJ ternary complex may gate the secretion of later T3SS substrates, such as harpins and effectors, pending additional secretion-inducing signals.

The HrpA1 pilus protein, described in Section 1.2 for its role as a positive regulator of the T3SS cascade, is also subject to post-translational regulation. The HrpA1 protein is rapidly degraded within the bacterial cell but can be stabilized through binding by T3SS regulatory protein HrpF [70,71]. HrpF is critical for disease and the production of a functional T3SS by various *P. syringae* strains [37,71], suggesting that HrpF stabilization of HrpA1 may be important for T3SS biogenesis. HrpF has also been shown to function as a negative regulator of the T3SS through interaction with HrpG [71]. Binding by HrpF may titrate HrpG from HrpG–HrpV complex formation, enabling HrpV to migrate away from the membrane and repress HrpS. The authors of this study proposed that the interaction of HrpF with HrpA1 during the early stages of T3SS production may reduce HrpG–HrpF interaction while favoring HrpG–HrpV complex formation, de-repressing HrpS and amplifying the expression of the T3SS signaling cascade. Further resolution of the HrpVGJ circuit, and its potential interplay with the intracellular dynamics of HrpF and/or HrpA1, will be a key part of defining the regulatory relationship between T3SS expression and secretion. 

The HrpA1 protein also accumulates extracellularly prior to the secretion of other T3SS substrates, including harpins and effector proteins [54,70]. In one study, an increase in the copy number of *hrpL* transcripts leads to increased extracellular accumulation of HrpA1 alone, while T3SS harpin and effector proteins accumulate intracellularly but were not secreted in higher amounts [54]. These findings suggest that the induction of the T3SS by *P. syringae* occurs in two discrete phases: (1) HrpL-dependent expression of T3SS genes and secretion of HrpA1, and (2) secretion of downstream T3SS substrates, which is likely regulated at the post-translational level. The means by which downstream T3SS substrate secretion is “unlocked” have not been identified, though the maturation of the HrpA1 pilus and eventual contact with the host plasma membrane may trigger secretion [70].

## 2. Environmental Regulation of the *P. syringae* T3SS

Despite its central role in virulence, the T3SS is not constitutively expressed by *P. syringae*. Therefore, *P. syringae* must devote significant cellular energy towards the production of the T3SS [72], and accomplish this process rapidly in order to overcome the host immune response. As such, a race between the deployment of the bacterial T3SS and host immunity defines the early stages of plant host infection by *P. syringae*, with the events taking place in the initial hours of infection critical in determining the long-term outcome and severity of disease development. Characterization of the host signals that induce T3SS deployment, and the mechanisms by which these stimuli are perceived in *P. syringae*, are key to understanding this sensitive and pivotal stage of infection.

### 2.1. Induction of the P. syringae T3SS in Response to Synthetic Media Conditions

Initial experiments to assess the regulation of the T3SS revealed that *hrp*/*hrc* genes are repressed when *P. syringae* are cultured in nutrient-rich media [42,73,74,75,76]. Various genes within the *hrp*/*hrc* pathogenicity cluster, including *hrpV* and *hrpT*, are required for maintaining repression of the T3SS in rich media, suggesting a low level of *hrp*/*hrc* expression occurs even under repressive conditions [36,77]. Based on a model proposed by Ortiz-Martín et al., this basal level of expression may “prime” the T3SS for rapid activation upon the removal of repressive environmental conditions [57,77]. In contrast to repression by rich media, *hrp/hrc* genes are induced when *P. syringae* are cultured in defined minimal media [73,78]. These media, variously referred to as *hrp*-inducing minimal medium (HIM or HMM), *hrp*-de-repressing medium (HDM) and/or inducing medium (IM), are characterized by a slightly acidic pH, a low nitrogen-to-carbon ratio, and the presence of a simple sugar such as fructose or mannitol as a carbon source. It should be noted that slight variations exist in the composition and pH of *hrp*-inducing minimal media described in various published works. Relative to a nutrient-rich medium such as King’s B, these minimal media conditions more closely mirror general features of the environment within the leaf apoplast. Together, these early findings suggested that T3SS-associated gene expression may be stimulated by general conditions encountered by *P. syringae* during infection rather than by specific plant signals.

### 2.2. Induction of the P. syringae T3SS by Specific Plant-Derived Signals

Although *hrp*/*hrc* genes are induced by synthetic media conditions, multiple studies observed comparatively higher levels of *hrp*/*hrc* expression during plant infection [42,53,70,75], suggesting the presence of additional T3SS-inducing signals specific to the plant environment. In early studies, the induction of most *hrp/hrc* genes including *hrpL* occurred during *P. syringae* infection of both resistant and susceptible cultivars of host plants [75], as well as during incompatible interactions with nonhost plants [42,79], indicating that the T3SS may be stimulated by signals common among plants rather than host-specific factors. In a study by Haapalainen et al., T3SS gene expression was assessed in *P. syringae* pv. tomato DC3000 cultured in a phosphate buffered-minimal medium supplemented with cell-free exudates from tomato cell suspension culture. A ten-fold increase in promoter activity of *hrpA1*, encoding the T3SS pilus protein, was observed in a minimal medium containing tomato exudates relative to phosphate buffer alone, suggesting that soluble plant signals were capable of eliciting T3SS gene expression [70]. Similarly, cell-free exudates from Arabidopsis suspension cell cultures were later observed to induce the expression of *hrpL* as well as the T3SS effector *avrPto* [80,81]. Co-culture of *P. syringae* with Arabidopsis suspension cells provides a tractable model system for measuring the abundance of plant T3SS-inducing metabolites and their effects on *P. syringae* virulence. Using this “infection-in-a-flask” system, a recent study discovered that the growth of *P. syringae* pv. tomato DC3000 was induced in the presence of Arabidopsis cells [80]. This effect was dependent on the production of a functional T3SS by *P. syringae*, suggesting that plant-derived signals stimulated bacterial growth by enhancing T3SS deployment [80].

Recent progress has been made in identifying specific metabolites in plant exudates that induce T3SS during infection. In one study, plant exudates prepared by soaking Arabidopsis seedlings in water were found to strongly induce *hrpL* expression and AvrPto accumulation in *P. syringae* pv tomato DC3000 [82]. A metabolomics analysis of these seedling exudates, followed by functional testing of the individual compounds identified, revealed multiple organic acids and amino acids, including citric acid and aspartic acid, as the bioactive T3SS-inducing compounds. Citric acid and aspartic acid are likely abundant in the tissues of all terrestrial plants, supporting previous observations that the *P. syringae* T3SS is induced by universal features of the plant environment, rather than host-specific signals [83,84]. Citric acid was also recently identified as a bioactive T3SS-inducing compound in Arabidopsis suspension cell exudates [80]. Although all of the bioactive compounds have carboxyl groups and are acidic, not all carboxyl-containing compounds present in exudates, for example, the amino acids leucine and valine are able to induce T3SS, indicating some level of specificity in their detection by *P. syringae*. Additionally, as described in more detail below, the maximal bioactivity of the T3SS-inducing acidic metabolites requires the presence of a simple sugar such as fructose, suggesting that *P. syringae* may rely on multiple distinct chemical cues during infection. Simple sugars have also been shown to increase phytotoxin production by *P. syringae* pv. *syringae* B3AR132 in response to plant phenolic glucosides [85]. Together, these findings indicate that simple sugars such as fructose may “prime” the *P. syringae* response to other plant compounds that induce virulence-related phenotypes.

The importance of specific plant-derived T3SS-inducing signals in determining *P. syringae* infection outcomes was demonstrated through studies of an Arabidopsis mutant plant lacking the immune regulator *MAPK PHOSPHATASE 1* (*MKP1*) [82]. The *mkp1* mutant was reported to have a heightened immune response to pathogen-derived elicitors and to be more resistant to *P. syringae* DC3000 infection [86]. Furthermore, during infection of *mkp1*, DC3000 had reduced ability to induce T3SS-associated genes and deliver effectors. GC-MS analysis of *mkp1* exudates revealed lower levels of multiple T3SS-inducing metabolites including citric acid and aspartic acid [82]. In this same work, exogenous addition of these metabolites to *mkp1* plants restored the ability of DC3000 to deliver effectors and completely restored the susceptibility of *mkp1* to DC3000 infection. Together, these results revealed that the abundance of virulence-inducing signals present during infection is dependent on the plant host genotype and that these chemical signals are important determinants of the outcome of *P. syringae*–host interactions.

### 2.3. Negative Regulation of T3SS Gene Expression by Plant-Derived Compounds

Plant-derived compounds capable of inhibiting T3SS expression by *P. syringae* have also been identified. One such T3SS inhibitor is auxin, or indole-3-acetic acid (IAA), a plant hormone that is also synthesized by *P. syringae* [87,88,89]. Auxin suppresses T3SS-associated gene expression in *P. syringae,* while stimulating the expression of genes encoding the type IV secretion system (T6SS), which is involved in microbial competition [88,89]. As IAA accumulates in *P. syringae*-infected leaves [90,91,92], T3SS suppression by IAA may enable *P. syringae* to taper T3SS production in the advanced stages of infection after host immune defenses have been sufficiently disarmed and bacterial growth established in the apoplast [88]. In this way, *P. syringae* may maximize its fitness by shifting cellular resources from the production of the T3SS to the T6SS during the later stages of infection, due to increased bacterial density in the apoplast and thus greater potential for microbial competition [88,93]. The expression of T3SS-encoding genes in *P. syringae* pv. tomato DC3000 is also inhibited by sulforaphane, a defense-associated glucosinolate produced in Arabidopsis leaves and in other Brassicaceae [94]. Sulforaphane suppresses T3SS gene expression through direct covalent modification of T3SS master regulator HrpS. Arabidopsis plants deficient in glucosinolate production exhibited increased susceptibility to *P. syringae* infection, suggesting that T3SS inhibition by sulforaphane may be an important layer of host defenses [94]. Other plant-derived compounds including plant flavonoids and other phenolics [95,96,97], as well as a variety of synthetic compounds [98,99], have also exhibited inhibitory activity towards the *P. syringae* T3SS.

### 2.4. Dynamics of T3SS Deployment within the Host Plant Environment

Multiple distinct approaches have been taken to assess the deployment of the T3SS by *P. syringae* during infection. In early work, pre-treatment of *P. syringae* with RNA or protein synthesis inhibitors prior to infection prevented *P. syringae* from eliciting T3SS effector-dependent plant defenses, providing foundational evidence that the expression of T3SS-encoding genes is necessary at early stages of infection [100]. In planta expression of T3SS genes was also assessed using promoter reporter fusions and the quantification of T3SS-encoding transcripts [53,79]. These studies revealed that T3SS genes are rapidly induced within the initial hours of plant infection, with transcript accumulation detectable as early as two hours post-inoculation into host leaves [100]. Transcript levels of *hrpR* accumulated more rapidly than other T3SS genes during host infection, consistent with the role of HrpR as an upstream regulator of the T3SS cascade [42,53]. More recently, various global transcriptomic analyses of *P. syringae* within the plant environment have similarly observed the induction of T3SS gene expression during the initial hours of infection [101,102,103]. Interestingly, a general decline in T3SS expression in *P. syringae* has been observed after the initial 24 h of host infection, suggesting complex temporal regulation may occur [93].

T3SS deployment by *P. syringae* may also vary between distinct plant microenvironments inhabited during infection. A transcriptomic analysis of *P. syringae* pv. *syringae* B728a cells extracted from either the leaf surface or apoplast of bean plants indicated that the T3SS was expressed to a greater extent within apoplastic sites of host leaves [102]. While the underlying cause of this difference is not known, cuticle wax components were shown to inhibit T3SS expression by both *P. syringae* pv. *phaseolicola* NPS3121 and *P. syringae* pv. tomato DC3000 and therefore could contribute to this phenotype [79]. Within the plant leaf apoplast, *P. syringae* exhibited maximal T3SS expression within the substomatal chamber, which is typically the first location colonized by infectious bacteria [79]. Although T3SS genes were expressed by *P. syringae* on the leaf surface, the role of the T3SS in epiphytic fitness remains ambiguous. In a genome-wide fitness profiling study in *P. syringae* pv. *syringae* B728a, genes encoding the T3SS provided a greater fitness benefit within the foliar apoplast than on the surface of host plants [104]. However, in other work the T3SS promoted epiphytic survival of *P. syringae* pv. *syringae* B728a on host leaf surfaces, with T3SS effectors HopZ3 and HopAA1 specifically enhancing epiphytic bacterial growth [105,106].

Within the host apoplast, *P. syringae* reversibly differentiates into subpopulations of high- and low-T3SS expressing cells [107,108]. This phenotypic differentiation required the HrpVG negative regulatory circuit, and was enhanced by the HrpA1 T3SS pilus protein, potentially due to its role as a positive auto-activator of the T3SS [107,108]. Bistable (stable high and low) levels of T3SS gene expression have been noted in a variety of Gram-negative bacterial pathogens [109,110], though the biological significance of this phenomenon is not fully understood [66,107]. Given that the production and maintenance of the T3SS is energetically costly [72], low-T3SS expressing “cheater” cells may be free to devote their energetic resources to multiplication or other cellular processes, promoting the overall resiliency and growth potential of the pathogen population [110]. The consequences of this behavior for *P. syringae* virulence, as well as the specific mechanism(s) by which T3SS bistability is initiated and maintained in the host environment, have yet to be fully characterized.

## 3. Molecular Mechanisms of T3SS Deployment in Response to Environmental Stimuli

While horizontal acquisition of pathogenicity islands such as the T-PAI is a common occurrence in the evolution of bacterial pathogens, additional evolutionary events are often needed to integrate newly acquired genetic material into existing regulatory networks [111,112,113]. It is worth noting that T3SS regulators HrpR and HrpS belong to the NtrC-like enhancer-binding protein family, yet lack a canonical receiver domain and are unlikely to directly perceive T3SS-inducing signals [56,114]. Furthermore, proteins predicted to function as receptors for extracellular signals are not encoded within the T-PAI. Therefore, environmental regulation of the *P. syringae* T3SS is likely due to factors encoded outside of the T-PAI. As described in more detail below, the emerging picture is that host signal-dependent regulation of type III secretion is complex, with multiple independent signaling pathways that sense and respond to distinct host signals acting in combinatorial fashion to effect T3SS deployment. Although how *P. syringae* perceives T3SS-inducing environmental signals is still poorly understood compared to other aspects of *P. syringae* pathogenesis, in this section we highlight recent progress in identifying signaling components that link distinct environmental signals directly to the regulation of the canonical HrpR/HrpS–HrpL cascade.

### 3.1. AauS-AauR Directly Regulates hrpRS Expression in P. syringae in Response to Specific Host-Derived Amino Acids

**Figure 2:**
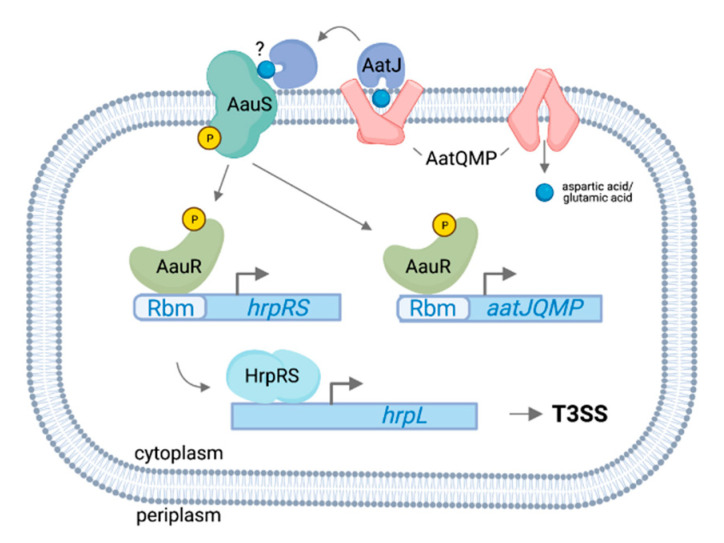
Model of regulation of the T3SS in *P. syringae* by AauSR. Depicted is a *P. syringae* cell, with only the inner membrane (separating the periplasm and cytoplasm) shown for clarity. In the periplasm, acidic amino acids such as aspartic and glutamic acid are bound by periplasmic solute binding protein AatJ (mauve). AatJ assists transport of these amino acids into the cytoplasm through interaction with the AatQMP ABC transporter (pink), and may additionally interact with the inner membrane histidine kinase AauS (teal). AauS activates its cognate response regulator AauR (olive) through phosphorelay. In its active conformation, AauR stimulates expression of the *aatJQMP* operon through binding of a conserved AauR binding motif (Rbm) in the *aatJ* promoter. AauR additionally induces *hrpRS* expression through binding an Rbm in the *hrpRS* promoter. HrpRS (aqua) then bind the enhancer of T3SS master regulator *hrpL*, leading to activation of the T3SS cascade. Figure was made using BioRender (https://app.biorender.com, accessed on 30 April2021).

Two-component systems are commonly involved in the environmental regulation of bacterial virulence [115,116]. These regulatory systems consist of a sensor histidine kinase, often localized to the bacterial membrane, and an intracellular response regulator that functions as a transcription factor to modulate gene expression in response to the activating stimulus [117]. In a recent Tn*5* genetic screen in *P. syringae* pv. tomato DC3000, genes encoding the Acidic Amino Acid Utilization Sensor and Response Regulator (AauSR) two-component system were identified as required for T3SS gene expression in response to fructose and aspartic acid [118]. AauS and AauR are encoded within the conserved *aat/aau* locus that also includes genes for an ABC transporter (AatQMP), as well as a periplasmic substrate-binding protein (AatJ). The *aat*/*aau* locus was first characterized in nonpathogenic *Pseudomonas putida* [119,120]. In *P. putida*, AatQMP and AatJ function in the cellular uptake of aspartic acid and glutamic acid, whereas AauS perceives the extracellular accumulation of these transport substrates, with activated AauR in turn binding to a conserved AauR-binding motif (Rbm) upstream of *aatJ* to upregulate the expression of an *aatJQMP* operon. In *P. syringae* DC3000, *aauS*, *aauR* and *aatJ* were each required for maximal expression of *hrpRS* and *hrpL* in response to aspartic acid or glutamic acid [118]. Furthermore, *aauSR* and *aatJ* were required for full virulence of DC3000 on host plants. By contrast, *aatQMP* was not required for T3SS expression in response to acidic amino acids, demonstrating that substrate uptake and T3SS signaling functions of the *aat/aau* module are genetically separable. To determine how the *aat*/*aau* locus regulates T3SS, the authors searched the DC3000 genome for Rbm sequences and identified an additional Rbm upstream of *hrpRS*, suggesting possible direct regulation of *hrpRS* expression by AauR (Figure 2). Indeed, deleting the Rbm upstream of *hrpRS* resulted in decreased T3SS expression in response to aspartic acid and glutamatic acid, as well as decreased DC3000 virulence on Arabidopsis [118]. The Rbm in the *hrpRS* promoter is conserved across 38 isolates of *P. syringae* containing the canonical T-PAI, indicating broad functional conservation of AauSR regulation of *hrpRS*. Consistent with this observation, *aauR* in *P. syringae* pv. *syringae* B728a was also required for maximal T3SS expression and virulence of B728a on host bean plants [118]. Based on these collective data, the authors hypothesized that the acquisition of Rbm upstream of *hrpRS* occurred early in *P. syringae* evolution as a means to connect the perception of environmental signals to the regulation of genes within the T-PAI. Given that aspartic acid and glutamic acid are among the most abundant amino acids in plants [84], the integration of T3SS expression with a pre-existing mechanism of amino acid sensing was possibly a critical early step in the evolution of *P. syringae* virulence.

### 3.2. The DeoR-Type Regulator SetA Is Necessary for hrpL Expression in Response to Sugar Signals

Simple sugars such as sucrose and glucose are abundant plant metabolites present within the leaf apoplast and exuded onto leaf surfaces [83,84]. As such, they are likely among the first metabolites encountered by *P. syringae* during infection. During culturing of *P. syringae*, the addition of a simple sugar to the culture medium is sufficient to induce T3SS gene expression [83]. Furthermore, maximal activation of T3SS genes by organic acids and amino acids in plant exudates, as described in Section 2.2 above, also requires the presence of sugars in the induction medium. Together, these observations suggest sugars may be important signals for *P. syringae* to initiate infection. In a recent Tn*5* mutagenesis screen, two *P. syringae* DC3000 mutants were recovered that contained insertion mutations in a gene encoding SetA (for Sugar-Induced Expression of T3SS A), a predicted DeoR-type transcription factor [121]. Phenotypic analysis of these mutants revealed that SetA was partially required for DC3000 expression of *hrpL* and effector *avrRpm1* in response to multiple sugars and sugar alcohols such as sucrose, fructose and mannitol, as well as maximal *hrpL* expression within the leaves of host Arabidopsis plants [121]. In this study, a DC3000 *setA*::Tn*5* mutant was unable to grow to high levels and elicit disease symptoms on infected host plants, likely due to impaired activation of its T3SS. Although *hrpL* expression was compromised in DC3000 *setA*::Tn*5*, the expression of upstream regulators *hrpRS* and *rpoN* was not altered, suggesting SetA may function at the level of *hrpL* expression or post-transcriptional regulation of HrpRS and/or RpoN [121]. Given that DeoR-type proteins typically function as DNA-binding transcriptional repressors, SetA may function by inhibiting the expression of a negative regulator of *hrpL.* In addition to its DNA-binding domain, SetA also possesses a C-terminal sensor domain that, based on other DeoR-type regulators, likely binds specific intracellular metabolites and regulates the DNA-binding activity of SetA. While sugar-induced expression of *hrpL* was partially SetA-dependent, DC3000 growth in media containing sugars as a sole carbon source did not require SetA, indicating that sugar-induced expression of the T3SS is genetically separable from sugar catabolism. These observations suggest SetA may function in regulating T3SS genes by sensing and responding to the intracellular accumulation of sugars or associated downstream catabolic products. Further characterization is necessary to identify the targets of SetA transcriptional regulation, as well as ligands that may bind to and module SetA activity.

### 3.3. Negative Regulation of the T3SS by the GacSA Global Regulatory System

The GacSA two-component system is conserved throughout Pseudomonas spp. and has been broadly characterized for its pleiotropic roles as a regulator of secondary metabolism and host–microbe interactions [122]. In this two-component system, the inner membrane-localized sensor kinase GacS responds to an unknown stimulus and activates cytoplasmic response regulator GacA by phosphorelay [122,123,124,125,126]. The GacSA system was initially identified as a regulator of *P. syringae* virulence over 30 years ago. In this work, isolates of *P. syringae* pv. *syringae* B728a containing insertion mutations in *gacS* were identified for their inability to cause disease lesions on host bean plants [127]. Based on these observations, the GacS sensor kinase in *P. syringae* was originally named LemA (for Lesion Manifestation A). The authors were unable to identify a cognate response regulator in the genomic neighborhood of *gacS*, despite two-component systems being typically encoded together within operons in bacterial genomes [127,128,129]. GacS was later genetically determined to form a two-component system in *P. syringae* with FixJ-like response regulator GacA, similar to the BarA/UvrY two-component system in *Escherichia coli* [123,125,130]. Throughout the γ-proteobacteria, *gacA* is located distally from *gacS* and shares an operon with *uvrC*, encoding a component of the nucleotide excision repair complex, though the functional significance of this pairing is unclear [122,131].

The role of GacSA as a regulator of the T3SS in *P. syringae* has been clouded by conflicting experimental results. While some studies concluded that GacSA positively regulates T3SS expression in *P. syringae* [63,95,132], other studies reported that GacSA negatively regulated T3SS expression, and was dispensable for T3SS deployment in the plant environment [127,133]. In contrast to conflicting *P. syringae* studies, GacSA was identified as a negative regulator of the T3SS in the animal pathogen *Pseudomonas aeruginosa* [134,135,136]. The ambiguous role of GacSA in *P. syringae* has complicated the development of cohesive models of GacSA function and T3SS regulation among all *Pseudomonas* pathogens. An isolate of *P. syringae* pv. tomato DC3000 containing a Tn*5*::*gacA* insertion, termed AC811, was studied to determine the molecular basis of GacSA regulation of virulence [63]. The authors demonstrated that the AC811 mutant was attenuated in both virulence and production of the T3SS in the plant environment. Largely based on this study, the role of GacSA as a regulator of virulence through positive control of the T3SS became the prevailing model in *P. syringae*, reflected in various contemporary reviews [115,137,138] and mathematical models of virulence regulatory networks in *P. syringae* [139].

Contrary to this early model, evidence has recently emerged to suggest that GacSA functions as a negative regulator of T3SS in *P. syringae*. A re-evaluation of the DC3000 *gacA*::Tn*5* isolate AC811 and other DC3000 *gacA*^-^ loss-of-function mutants revealed that the inactivation of *gacA* enhanced, rather than reduced, T3SS gene expression. Furthermore, this study demonstrated that *gacA* was not required for *P. syringae* T3SS deployment and virulence within the host foliar apoplast [140]. Rather, attenuated virulence of the *gacA*::Tn*5* AC811 strain was attributed to a second-site mutation in *anmK*, an enzyme involved in bacterial cell wall recycling, as well as negative polar effects of the *gacA*::Tn*5* insertion on the transcription of downstream gene *uvrC* [141]. While neither *anmK* nor *uvrC* were previously associated with *P. syringae* virulence, a polar effect of a *gacA* mutation on the expression of downstream *uvrC* was reported to impact the virulence of the closely related animal pathogen *Pseudomonas aeruginosa* [142,143].

**Figure 3:**
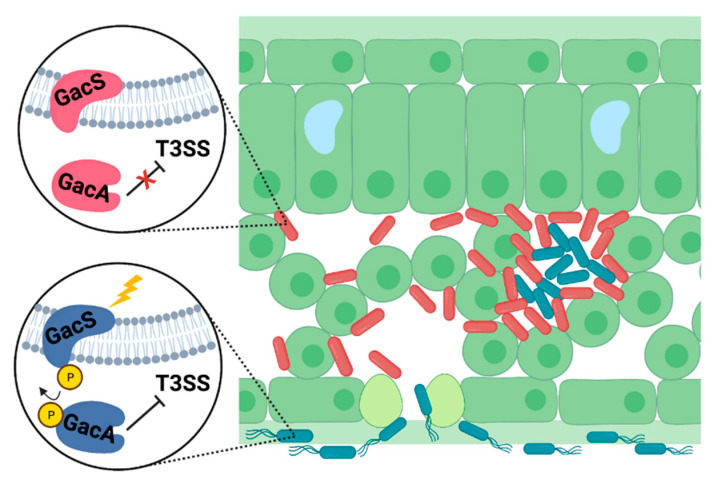
Hypothetical model of GacSA-mediated repression of the T3SS in *P. syringae* infection. Depicted is a leaf cross-section infected with *P. syringae*. Cells with an activated GacSA system are in blue (lower inset; GacA-P^+^), while cells with an inactive GacSA system are in red (upper inset; GacA-P^-^). On the leaf surface (lower leaflet), the GacSA system is activated by an unknown stimulus (lightning bolt), leading to repression of the T3SS and promotion of flagellar motility. GacA-P^+^ cells use flagellar motility to infiltrate gaps in the leaf surface, gaining access to the leaf interior or apoplast. GacSA is deactivated within the apoplast, leading to de-repression of the T3SS. GacA-P^-^ cells deploy the T3SS in order to disarm host immune defenses and establish *P. syringae* growth in the apoplast. Once *P. syringae* has grown to a high density within the apoplast in the advanced stages of infection, GacSA may be re-activated to downregulate T3SS expression in order to conserve cellular energy. This model of GacSA regulation is based solely on phenotypes of *gacA^-^* loss-of-function mutants and is not yet supported by biochemical evidence. Figure was made using BioRender (https://app.biorender.com, accessed on 30 April2021).

In contrast to T3SS deployment, GacA was required for the cellular motility of DC3000, suggesting that the T3SS and motility may be subject to inverse regulation in *P. syringae* [63,95,140]. The requirement of GacSA for motility may explain why various *P. syringae gacS^-^* and *gacA^-^* mutant isolates are unable to effectively colonize host plants, as bacterial motility is often required for epiphytic *P. syringae* to gain access to the foliar apoplast through stomata or other openings in the leaf surface [127,140]. Together, these results support a model in which GacSA is activated on the leaf surface, where it suppresses the T3SS and induces cellular motility, potentially promoting *P. syringae* epiphytic fitness and ingress into the leaf interior. GacSA is then deactivated within the apoplast, permitting de-repression of the T3SS (Figure 3). Future characterization of where and when during plant infection the GacSA system is activated, as well as the identification of the GacSA inducing signal(s), is necessary to further evaluate this model. The mechanism(s) by which GacSA affects T3SS deployment is also not clear, though GacSA is known to modulate the activity of the RsmA regulatory protein family reviewed in Section 4.4.

### 3.4. Negative Regulation of the T3SS by the RhpSR Two-Component System

The RhpSR two-component system, characterized primarily in the model pathogen *P. syringae* pv. *phaseolicola* 1448A (alternatively *P. savastanoi* pv. *phaseolicola* or *P. amygdali* pv. *phaseolicola*), also regulates the expression of T3SS-associate genes. In its phosphorylated form, the response regulator RhpR represses the T3SS through binding to a semi-conserved inverted repeat element in the promoters of *hrpR* and T3SS effector gene *hopR1* [144,145,146]. Phosphorylated RhpR additionally downregulates the T3SS through the activation of the promoter of the *lon* protease gene, promoting Lon-mediated HrpR degradation [144,145,146,147]. The sensor histidine kinase of this two-component system, RhpS, is capable of autokinase activity, and exhibits both kinase and phosphatase activity towards RhpR [146]. Under T3SS-repressive environmental conditions, such as nutrient abundance in rich culture medium, RhpS functions as a kinase to promote T3SS repression through the phosphorylation of RhpR, while T3SS-inducing conditions favor RhpS phosphatase activity towards RhpR, thereby decreasing RhpR activity at target promoters [144,146].

RhpR is also phosphorylated independently of RhpS, potentially by physiological phosphodonors such as acetyl phosphate and/or by sensor kinases from other two-component systems [146]. In addition to the repression of the T3SS, RhpR broadly regulates central cellular maintenance, including ribosomal protein synthesis and various cell envelope-related processes, in a phosphorylation state- and culture medium-dependent manner [144,146,148]. RhpR similarly activates its own promoter, thereby maintaining T3SS repression under non-permissive conditions by a negative feedback loop [146,147]. Co-regulation of T3SS and cellular maintenance by the RhpSR system may facilitate a general tradeoff between pathogen virulence and metabolism according to environmental conditions such as nutrient availability [145,146]. However, the specific environmental stimuli that govern RhpSR activity remain unknown.

### 3.5. Regulation of the T3SS by the CvsSR Two-Component System

The calcium-induced two-component system CvsSR is a regulator of *P. syringae* T3SS gene expression and virulence [149]. The expression of CvsSR is induced in DC3000 treated with apoplastic fluid from tomato leaves, and is also induced by various cations including calcium (Ca^2+^) [149,150]. Calcium is abundant in the plant apoplast and has been observed to increase in concentration during bacterial infection of host plants [151,152]. In T3SS-inducing minimal medium supplemented with calcium, response regulator CvsR binds the *hrpRS* promoter as well as various sites in or around T3SS effector genes, and drives the expression of T3SS-associated genes [149]. Loss-of-function *cvsS^−^* and *cvsR^−^* mutations have been observed to attenuate DC3000 growth and disease symptom formation on host plants, indicating a role of CvsSR in overall *P. syringae* virulence [149]. However, CvsSR was not required for T3SS deployment by DC3000 in *Nicotiana benthamiana* [149]. As such, it remains unclear whether CvsSR contributes to virulence through the regulation of the T3SS in the host environment. CvsSR additionally indirectly represses the expression of *algU*, an alternate sigma factor that functions as a positive regulator of the T3SS in DC3000 by an unknown mechanism (reviewed in Section 4.2), indicating potentially complex effects of CvsSR on T3SS dynamics [149].

## 4. Additional Global Regulators of the T3SS in *P. syringae*

Bacteria often perceive and respond to extracellular stimuli through a network of intracellular signaling pathways, providing multiple potential points of regulation and signal amplification [153]. In addition to putative receptors such as AauSR and RhpSR that directly regulate T3SS genes in response to environmental signals, additional proteins and small RNAs in *P. syringae* that modulate T3SS expression, most likely through indirect mechanisms, have been identified. In addition to tuning responses to host signals, in some cases these regulators also facilitate regulatory crosstalk between the T3SS and other cellular processes that are potentially influenced by T3SS activation, including central metabolism or cellular motility [60,63,140]. In this section, we highlight additional regulators that are known to influence the deployment of T3SS regulators HrpRS and HrpL.

### 4.1. Dual Positive and Negative Regulation of the T3SS by Bifunctional Lon Protease

Lon is an ATP-dependent protease and a DNA-binding transcription factor [154,155]. As a protease, Lon suppresses T3SS expression by degrading HrpR, thereby attenuating the expression of *hrpL* and downstream genes in the T3SS regulon [77,156,157,158]. Lon may also suppress the T3SS by degrading effector proteins prior to secretion, a process counteracted by interactions between T3SS effectors and their cognate chaperones [159,160]. As a transcription factor, Lon positively autoregulates its own expression under T3SS-restrictive conditions by binding its own promoter [155]. Early evidence suggested Lon is expressed constitutively in *P. syringae* as it is in other bacterial species [57,160]. Under this model, constitutive proteolytic activity by Lon was predicted to be sufficient to suppress HrpR activation of the T3SS under conditions where the expression of HrpR is low, such as in rich media. When *hrpR* transcript levels rapidly spike upon exposure of *P. syringae* to T3SS-inducing signals, the enhanced levels of HrpR are presumably beyond the threshold of effective suppression by Lon, permitting de-repression of T3SS [53,60]. However, more recent studies have determined *lon* promoter activity to be positively regulated by RhpR(-P), a T3SS repressor, and stimulated in the presence of acetate [144,148]. Furthermore, Lon expression is elevated in T3SS-deficient mutants of *P. syringae*, suggesting the presence of an as-of-yet uncharacterized negative feedback loop that suppresses Lon following T3SS activation [148].

Regulation of the T3SS by Lon is complex and multi-factorial, particularly given that Lon functions as a pleiotropic regulator in *P. syringae*. As a transcription factor, Lon regulates the expression of various global regulatory systems in *P. syringae*, and therefore may indirectly participate in T3SS regulation [155]. *Lon^-^* loss-of-function mutants of *P. syringae* and other bacteria generally exhibit attenuated stress tolerance, and have also been observed to elicit a stronger immune response from host plants during infection [154,156,158]. While various studies have noted that Lon is necessary for full virulence of *P. syringae* in host leaves [157,158], it remains unclear whether this virulence defect is related to the T3SS versus other components of the Lon regulon. In addition to its role as a negative regulator of T3SS, Lon may also positive regulate T3SS deployment under certain environmental conditions. Loss-of-function *Lon^-^* mutants of *P. syringae* pv. *phaseolicola* 1448A accumulated reduced levels of T3SS-associated gene transcripts, including upstream regulator *hrpRS*, when cultured in a T3SS-inducing minimal medium [155,157]. However, other studies have reported that Lon functions as a negative regulator of T3SS under both T3SS-restrictive and T3SS-inducing conditions [77,156,158]. Whether Lon functions as a positive regulator of T3SS in the plant environment, and/or how negative regulation by Lon may enable the fine-tuning of T3SS deployment during host infection, has yet to be fully characterized.

### 4.2. Regulation of the T3SS by Alginate Master Regulator AlgU

AlgU, an extra-cytoplasmic function (ECF) alternate sigma factor, also functions in the activation of the T3SS signaling cascade [161,162]. In some *P. syringae* pathovars, AlgU was required for full expression of T3SS-associated genes, though AlgU does not appear to regulate T3SS expression in other pathovars, suggesting the diversification of the AlgU regulon within the *P. syringae* species complex [103,161,162]. In *P. syringae* pv. tomato DC3000, an *algU^-^* loss-of-function mutant exhibited reduced expression of *hrpL* and *hrpRS*, and influenced the expression of a significant portion (38%) of the HrpL regulon [161,162]. AlgU additionally directed the expression of *algW*, a potential positive regulator of the T3SS identified in *P. syringae* pv. *maculicola* ES4326 [163]. The expression of AlgU was stimulated by a variety of environmental stressors, including osmotic stress and perturbation of the bacterial cell envelope; however, it is unknown whether these environmental stimuli also regulate the T3SS in *P. syringae* [164,165,166,167]. The expression of AlgU was also indirectly repressed by the calcium-induced CvsSR two-component system previously reviewed in Section 3.5 [149].

The functionally diverse regulon of AlgU provides some indication as to how *P. syringae* may coordinate the regulation of the T3SS with other phenotypes relevant to host infection. Per its nomenclature, AlgU stimulates the biosynthesis of alginate, an extracellular polysaccharide that plays a role in the virulence of select *P. syringae* strains on host plants [161,166,168,169,170]. Alginate may provide protection against cellular osmotic stress caused by plant immune defenses, and *algU* expression and alginate production by *P. syringae* are potential targets of the host immune response [171,172]. Additionally, the AlgU regulatory system is broadly implicated in negative regulation of the bacterial flagella, providing further evidence that the T3SS and flagellar motility may be subject to inverse regulation in *P. syringae* [161,163,173]. The production of coronatine, a phytotoxin involved in the regulation of host stomatal aperture, is also subject to regulation by AlgU [162]. These findings suggest that alginate and coronatine production may be activated concurrently with T3SS deployment during *P. syringae* infection, while flagellar motility is downregulated.

### 4.3. Regulation of the T3SS by AHL Quorum Sensing Regulator AefR

AefR, a TetR-like transcriptional regulator, may also function as a positive regulator of the T3SS by a yet unknown mechanism [174,175,176]. AefR was required for full virulence of several *P. syringae* strains, and variably contributed to epiphytic stress tolerance, invasion of the leaf interior, and/or surface motility in a strain-specific manner [174,175,177]. Initially identified for its role in acyl homoserine lactone (AHL) quorum sensing, AefR was also required for AHL production across multiple different *P. syringae* strains [174,175,176,178]. The loss of *aefR* in the bean pathogen *P. syringae* pv. *phaseolicola* NPS3121 reduced *hrpR* promoter activity in host leaves, yet did not entirely abolish the production of a functional T3SS [175]. However, a subsequent study in *P. syringae* pv. *tabaci* 6605 reported no contribution of AefR to T3SS gene expression [176]. Therefore, the role of AefR in T3SS regulation, as well as the potential intersection between T3SS and AHL quorum sensing in *P. syringae*, remain unclear. A transcriptomic analysis of the AefR gene regulatory network revealed a small AefR regulon that did not significantly impact T3SS-associated gene expression, although the results of this study did support the role of AefR as a genetic regulator of AHL quorum sensing in *P. syringae* pv. *syringae* B728a [103].

### 4.4. Modulation of T3SS Gene Expression by RsmA RNA-Binding Proteins

The RsmA protein family is implicated in the regulation of T3SS gene expression in *P. syringae*, as well as various traits related to plant–microbe interactions in *P. protegens* CHA0 (alternatively *P. fluorescens* CHA0), a nonpathogenic rhizosphere bacterium [63,179,180,181,182]. RsmA proteins are a family of regulatory mRNA-binding proteins that modulate the abundance of their target transcripts. The modulation of RsmA protein activity by the GacSA two-component system (reviewed in Section 3.3) has been extensively characterized in CHA0, and this regulatory network is often referred to as the Gac–Rsm system [182]. RsmA proteins can impact target mRNAs either negatively, i.e., by inhibiting the translation of bound mRNA transcripts, or positively, i.e., by stabilizing the target mRNA [183,184,185,186,187]. DC3000 possesses five distinct RsmA proteins (RsmA1–5), alternatively termed CsrA1–5 in some publications [132,188,189]. In a recent study, both RsmA2 and RsmA3 were required for full T3SS expression and virulence on host tomato [188,189]. Homologs of RsmA2 and RsmA3 were similarly required for virulence of *P. syringae* pv. *phaseolicola* 1448A [190]. However, a separate study of RsmA1–5 functions in DC3000 concluded that RsmA2 and RsmA3 were not required for T3SS deployment, and that RsmA3 appeared to function as a repressor of T3SS expression [132]. Additional experiments to resolve the exact mechanism(s) of T3SS regulation by RsmA2 and RsmA3 may be helpful to clarify these apparently conflicting findings.

In *Pseudomonas* spp., GacA neutralizes the regulatory effects of the RsmA protein family by directing the expression of the *rsm* family of small non-coding RNAs (ncRNAs) [181,191,192]. The *rsm* ncRNAs function as molecular sponges to titrate RsmA protein, preventing RsmA interaction with target mRNAs [179,180]. In DC3000, seven *rsm* ncRNAs (*rsmY*, *rsmX1-*5, and *rsmZ*) are expressed in a GacA-dependent manner [189,192]. Based on the recent characterization of GacA as a negative regulator of T3SS gene expression in DC3000, GacA-mediated expression of *rsmXYZ* may deactivate RsmA2 and/or RsmA3, preventing these regulatory proteins from activating T3SS expression [140,188,189]. However, this model conflicts with other experimental findings of RsmA function in DC3000, and has yet to be experimentally evaluated [132]. An Rsm-independent branch of the GacSA regulon may also exist [122,190], which may provide an alternative pathway to negative regulation of the T3SS.

### 4.5. Nucleotide Second Messengers in T3SS Regulation

Nucleotide second messengers are common intermediates in bacterial signal transduction. These compounds typically exhibit transient spikes in intracellular concentration under select conditions, effecting a temporally delimited response to an inducing stimulus [193]. One such messenger is (p)ppGpp, also referred to as the bacterial alarmone, a signal of bacterial amino acid starvation involved in broadly arresting cellular protein synthesis and redirecting energetic resources towards survival [194]. The synthesis of (p)ppGpp is required for *P. syringae* pv. *syringae* B728a to fully express T3SS genes, including master regulators *hrpL* and *hrpRS*, and is also required for virulence and epiphytic survival on host plants [195]. Another nucleotide second messenger, cyclic diguanylate (c-di-GMP), additionally influences the T3SS in *P. syringae*. High intracellular levels of c-di-GMP have been observed to suppress T3SS expression in *P. syringae* pv. *syringae* B728a, while enhancing oxidative stress tolerance [196]. In *P. syringae* pv. tomato DC3000, the gene encoding a c-di-GMP synthase termed Chp8 is regulated by HrpL and contributes to the virulence and tolerance of host immune defenses by an unknown mechanism [197]. While the role(s) of c-di-GMP in *P. syringae* virulence is(are) not fully understood, intracellular levels of c-di-GMP may balance T3SS deployment with environmental stress tolerance, both of which are required for the pathogen to effectively survive in the host environment. High intracellular levels of c-di-GMP additionally suppress flagellar motility in B728a [196]. While other reports suggest that T3SS and flagellar motility may be inversely regulated in *P. syringae* [161,163,173], the inhibitory effects of c-di-GMP on both T3SS and motility suggest a complex regulatory relationship between these processes.

## 5. Conclusions and Future Directions

The deployment of the T3SS by *P. syringae* is a complex and environmentally attuned process. Given that the initial hours of *P. syringae* infection critically determine the eventual outcome of disease, the elucidation of these early host–pathogen signaling events is fundamental to understanding *P. syringae* pathogenesis as a whole. Key points highlighted in this review include:

- *P. syringae* rapidly deploys the T3SS during the initial hours of plant host infection, and primarily relies on the T3SS to establish growth within the host apoplast. T3SS deployment may be negatively regulated in later stages of infection, possibly as a means to maximize fitness in the host environment.
- T3SS expression by *P. syringae* is induced by specific organic acids and amino acids that are abundant in the plant environment. These T3SS-inducing plant metabolites require the presence of a simple sugar such as fructose for maximal bioactivity, suggesting *P. syringae* coordinates T3SS deployment by sensing multiple distinct host signals.
- The abundance of T3SS-inducing metabolites in the host environment is genetically regulated by the plant host and significantly impacts the progression of *P. syringae* disease, as evidenced by the enhanced disease resistance phenotypes of the *mkp1* mutant of Arabidopsis.
- AauSR, a two-component system in *P. syringae* associated with the uptake of acidic amino acids by the ABC transporter AatQMP, directly regulates the expression of T3SS genes in response to host-derived aspartic acid and glutamic acid signals. Additional proteins including RhpSR, CvsSR and SetA may also function as sensors for host signals leading to T3SS regulation. However, the specific signal(s) these proteins detect and the means by which these various response pathways interact to synergistically regulate the T3SS are unknown.

Despite significant progress in identifying T3SS-inducing signals and their putative receptors, many aspects of T3SS regulation during host infection are poorly understood. Important questions remaining include:

- Do all *P. syringae* detect and respond to the same T3SS-inducing metabolites? Molecular studies of T3SS induction have so far been limited to a small number of *P. syringae* strains. Broadening future analyses to include additional strains that represent the diversity of the *P. syringae* species complex will be necessary to fully evaluate the conservation of host-perception mechanisms.
- How many distinct host signals does a single strain of *P. syringae* respond to? Mutants lacking putative host signal receptors (e.g., AauSR, SetA) are only partially attenuated in virulence, suggesting that multiple input signals may additively or synergistically contribute to T3SS induction in *P. syringae*.
- Multiple regulatory systems in *P. syringae* are known to influence T3SS dynamics—how do these distinct pathways intersect and converge on T3SS regulation? A newly published analysis of gene regulatory networks in *P. syringae* (termed PSRnet) indicates that complex crosstalk occurs between multiple known virulence regulators [198].
- At what point(s) during *P. syringae* infection is T3SS deployment repressed, and why? While evidence suggests that T3SS expression may be downregulated in *P. syringae* on the leaf surface and/or during later stages of apoplast infection, the mechanisms responsible for T3SS repression during host infection are not fully understood. Moreover, the role T3SS repression may play in *P. syringae* virulence and life cycle remains ambiguous. Further elucidation of how the T3SS is co-regulated with other virulence-related processes in *P. syringae*, as well as the impact of T3SS deployment on bacterial cell homeostasis and growth, may help to elucidate the potential benefits of T3SS repression.
- How is deployment of the T3SS spatially regulated within the host environment? Additionally, how is T3SS deployment regulated on the population level during *P. syringae* infection? Experiments to date suggest that only a subpopulation of *P. syringae* cells may deploy their T3SS during infection. Is this heterogeneity in part due to variation in the abundance of inducing signals within plant tissues? Continued development of transcriptomic methods for profiling gene expression by *P. syringae* within the host environment, such as single cell RNA-seq, as well as fluorescence-based reporters for in planta detection of T3SS expression, will be necessary to fully address these questions.

## Data Availability

Not applicable.
